# Determinants of chronic disease patients' intention to use Internet diagnosis and treatment services: based on the UTAUT2 model

**DOI:** 10.3389/fdgth.2025.1543428

**Published:** 2025-08-01

**Authors:** Jing Zhao, Bei Li, Jianwei Sun, Xu Zeng, Jing Zheng

**Affiliations:** ^1^ Department of Biomedical Informatics, School of Life Sciences, Central South University, Changsha, China; ^2^ Shenzhen Health Development Research and Data Management Center, Shenzhen, China

**Keywords:** Internet diagnosis and treatment, chronic disease patients, UTAUT2, behavioral intention, China

## Abstract

**Background:**

Chronic diseases are a significant public health concern. Internet diagnosis and treatment services can effectively monitor chronic diseases and are vital for alleviating the healthcare system burden caused by these conditions. Distinguishing itself from prior investigations, this study focuses on the critical cohort of chronic disease patients and, building upon the UTAUT2 framework, introduces additional constructs such as trust and medical habits. It systematically examines the pivotal determinants influencing the acceptance and utilization of Internet diagnosis and treatment services among chronic disease patients in Shenzhen, China.

**Objective:**

This study centers on the population of chronic disease patients in Shenzhen, China, by developing a theoretical model to elucidate their behavioral intentions toward utilizing Internet diagnosis and treatment services. Employing empirical methods, the research identifies the key determinants that influence patients' acceptance and adoption of these services. Furthermore, based on the interactive mechanisms among these factors, targeted policy recommendations are advanced to enhance service utilization rates and optimize the quality of Internet diagnosis and treatment services.

**Methods:**

Guided by the theoretical framework, and informed by expert consultations and a preliminary survey, the questionnaire was meticulously designed and refined. Employing a five-point Likert scale, the survey investigated the usage patterns of Internet diagnosis and treatment services among chronic disease patients in Shenzhen, China, as well as the factors influencing their behavioral intention. Utilizing convenience sampling, a total of 823 valid responses were collected. Subsequent data analysis was conducted using SPSS 26.0 and AMOS 28.0 software, encompassing descriptive statistics and structural equation modeling. Furthermore, the Bootstrap method was employed to rigorously assess the mediating effects within the model.

**Results:**

The empirical findings reveal that: (1) Model validation indicates that performance expectancy (*β* = 0.151, *p* = 0.002), effort expectancy (*β* = 0.105, *p* = 0.022), social influence (*β* = 0.206, *p* < 0.001), price value (*β* = 0.138, *p* = 0.002), trust (*β* = 0.124, *p* = 0.003), and electronic health literacy (*β* = 0.184, *p* < 0.001) exert significant positive effects on the behavioral intention to use Internet diagnosis and treatment services. Conversely, perceived risk negatively influences behavioral intention (*β* = 0.094, *p* = 0.008), whereas the effect of medical habits on behavioral intention is not statistically significant (*p* > 0.05). (2) Performance expectancy partially mediates the relationships between effort expectancy, trust, electronic health literacy, and behavioral intention, while effort expectancy partially mediates the relationship between electronic health literacy and behavioral intention.

**Conclusion:**

Performance expectancy, effort expectancy, social influence, price value, trust, perceived risk, and electronic health literacy constitute the principal determinants shaping the behavioral intention of chronic disease patients to adopt Internet diagnosis and treatment services. Drawing on these findings, this study advances targeted policy recommendations aimed at optimizing user experience and fostering the sustainable, high-quality development of Internet diagnosis and treatment services within chronic disease management.

## 1 Introduction

In this study, “Internet diagnosis and treatment” refers to the provision of online consultations, remote health management, follow-up, and other services for patients with common or chronic diseases by physical medical institutions using the Internet and other related information technologies, primarily via Internet hospital platforms (1). Internet diagnosis and treatment constitutes the core of Internet healthcare. The widespread adoption of smartphones and the development of robust communication networks have transformed the Internet into a bridge that facilitates communication between patients and doctors, enabling consultations and inquiries across both time and space. The COVID-19 pandemic in 2020 further accelerated the development of Internet healthcare. By 2023, the market size of Internet healthcare in China had reached 310.2 billion yuan, reflecting an approximate year-on-year growth rate of 39%; this figure is expected to increase to 419 billion yuan by 2024 (2). Concurrently, the number of Internet hospitals has grown substantially, with 3,340 hospitals nationwide providing more than 100 million diagnosis and treatment visits per year. User adoption has also been rising continuously; as of December 2024, nearly 1.108 billion people in China were Internet users, of whom 418 million engaged in Internet healthcare—accounting for 37.7% of all Internet users (3). However, this also indicates that more than 62% of Internet users have never utilized Internet healthcare services.

Data on user scale and usage rates collected between December 2022 and December 2024 (3), along with related surveys (4–6), highlight a significant challenge for the development of Internet diagnosis and treatment services in China: relatively low awareness and utilization among the target audience. Zhang et al. (7) conducted a survey and found that only 10.96% of outpatient patients in Jinan had used Internet diagnosis and treatment services, while 45.29% had heard of them but had not used them, and 43.74% had never heard of them, indicating a low level of patient awareness. Wang et al. (8) discovered that 37.8% of older adult individuals had used Internet healthcare services, with only 16.9% using them independently. Similar studies have shown that only 15.61% of outpatient patients were aware of and had used Internet diagnosis and treatment services, 40.13% were aware but had not used them, and 44.27% had never heard of them. This indicates that the actual usage rate of Internet diagnosis and treatment services in China is relatively low, and the level of public awareness remains insufficient (9). This is markedly lower than in European and American countries such as Germany (10), the United States (11), and the United Kingdom (12). In Jordan, a survey by Murshidi et al. (13) found that approximately 50% of the population was aware of telemedicine, but only about 25% were familiar with its practical applications. Nevertheless, 67.8% of respondents expressed a willingness to use telemedicine. In Australia, a study by Thomas et al. (14) revealed that 88.3% of respondents had engaged in some form of medical consultation in the past 12 months, with 69.3% having used telemedicine services. Compared to 2023, the usage rate of Internet healthcare declined in 2024—a downward trend expected to continue in the coming years. The low utilization and acceptance of Internet diagnosis and treatment services among users with high medical needs represents a critical contradiction that must be resolved to foster further industry development. By identifying the key factors influencing users' behavioral intentions to use Internet diagnosis and treatment services, service providers can implement targeted measures to optimize their offerings and accelerate industry growth.

Chronic non-communicable diseases (referred to as “chronic diseases”) primarily include cardiovascular and cerebrovascular diseases, malignant tumors, chronic respiratory diseases, and diabetes. According to estimates by the World Health Organization, chronic diseases were responsible for at least 43 million deaths worldwide in 2021, accounting for approximately 75% of global mortality. Of these fatalities, nearly 18 million occurred among individuals aged 30–70 years. Notably, 73% of all deaths attributable to chronic diseases transpired in developing countries. Four primary categories—cardiovascular diseases, cancer, chronic respiratory diseases, and diabetes—collectively accounted for roughly 35 million deaths. Specifically, cardiovascular diseases led to 19 million deaths, cancer to 10 million, chronic respiratory diseases to 4 million, and diabetes to 2 million. In China, chronic diseases account for over 88% of total mortality and contribute more than 70% of the overall disease burden. Among individuals aged 60 and above, the prevalence of chronic diseases exceeds 78% (15, 16).

Internet diagnosis and treatment services have overcome temporal and geographic limitations by promptly addressing patients’ medical needs, ensuring continuity of care, and facilitating a shift away from traditional chronic disease management models. Chronic disease patients represent an important user group of these services. However, existing research on behavioral intentions regarding Internet diagnosis and treatment services predominantly focuses on the general public, hospital patients, and the older adult, while studies from the perspective of chronic disease patients remain limited. Furthermore, international research on Internet diagnosis and treatment services for chronic disease patients has mostly concentrated on platform construction (17, 18) and application effect evaluation (19, 20), with fewer analyses of the factors influencing behavioral intentions to use these services. Consequently, there is a significant gap at both the practical and theoretical levels. This study aims to address this gap by investigating the acceptance of and the factors influencing the use of Internet diagnosis and treatment services from the perspective of chronic disease patients in China. The study seeks to answer the following research question:

• What are the key factors influencing the acceptance and adoption of Internet diagnosis and treatment services among chronic disease patients in China?

The extended Unified Theory of Acceptance and Use of Technology (UTAUT2) serves as the theoretical framework for this study, which empirically examines the factors affecting the acceptance and adoption of these services among chronic disease patients in developing countries. Four external variables—trust, medical habits, perceived risk, and electronic health literacy—were incorporated into the model, and an empirical analysis was conducted to assess their influence on the behavioral intention to use Internet diagnosis and treatment services among patients in Shenzhen, China.

## 2 Literature review and theoretical hypotheses

In recent years, significant progress has been made in research on Internet diagnosis and treatment services within the domain of chronic disease management. Existing studies primarily focus on two key dimensions: first, the system development and functional optimization of healthcare platforms, and second, the evaluation of service applications and empirical studies. Scholars both domestically and internationally have developed various innovative diagnostic and therapeutic platforms tailored to the personalized management needs of chronic disease patients. For example, Quan (21) designed a mobile Internet-based chronic disease diagnostic system, which integrates functions such as electronic medical record management, data collection, intelligent diagnosis, online communication, and health education, thus facilitating the intervention and management of chronic diseases. Ren et al. (17) developed an intelligent cloud platform for managing chronic heart failure, which incorporates features such as smart wearable devices, monitoring indicators, traditional Chinese medicine (TCM) information, health education, and early warning notifications. This platform combines Western and Chinese medicine approaches to enhance patients' self-management abilities, improve their quality of life, and reduce readmission rates and mortality. Jindal et al. (22) developed a mHealth intervention for low-resource regions, which integrates management of chronic conditions, evidence-based clinical decision support, longitudinal health data, and automated short-messaging service to enhance medication adherence and follow-up visit for patients with hypertension and diabetes. Korpershoek et al. (23) employed a user-centered design (UCD) process to develop evidence-driven mobile health interventions specifically for patients with chronic obstructive pulmonary disease (COPD), enhancing self-management of disease exacerbation.

Studies have shown that health management through Internet diagnosis and treatment platforms can effectively improve patient outcomes. For instance, Wang et al. (24) found that patients with type 2 diabetes enrolled in the Cloud Hospital Health Management Platform significantly outperformed the traditional management group in terms of blood sugar control, BMI, and self-efficacy scores. Deng et al. (19) reported that after 6 months of managing hypertension patients via a mobile healthcare app, the experimental group showed superior treatment rates, blood pressure control rates, and self-management scores compared to the control group, with better results in blood pressure treatment. A systematic review by Koh et al. (25) indicated that telemedicine interventions for chronic obstructive pulmonary disease (COPD) were either as effective as or superior to conventional care. Felker et al. (20) found that a 3-month mobile health intervention significantly improved daily physical activity, health-related quality of life, and metabolomic markers of car-diovascular health in patients with heart failure and diabetes. These studies clearly demonstrate the significant advantages of Internet diagnosis and treatment platforms in disease monitoring, outcome improvement, and health management, highlighting the extensive application value of telemedicine in chronic disease management.

However, existing research still exhibits insufficient exploration of the behavioral mechanisms and adoption factors of chronic disease patients, areas that require further in-depth investigation and expansion. Existing research on the factors influencing the behavioral intention to use Internet healthcare services frequently employs the Technology Acceptance Model (TAM), the Unified Theory of Acceptance and Use of Technology (UTAUT), and other theoretical frameworks (26). TAM elucidates how perceived usefulness and ease of use shape users' adoption decisions (27). In 2003, Venkatesh et al. (28) synthesized eight theoretical models, including TAM, to propose the UTAUT model, which posits that users' willingness to use and their usage behavior are determined by performance expectancy, effort expectancy, social influence, and facilitating conditions. Empirical studies have demonstrated that the UTAUT model accounts for up to 70% of the variance in behavioral intention, outperforming each of the eight individual models (29). Zhang et al. (30) expanded TAM by incorporating external variables—privacy security, communication convenience, and technological ease of use—that affect perceived usefulness and ease of use, thereby exploring factors influencing patients' acceptance and use of Internet healthcare services. AlBar et al. (31) integrated TAM with the Theory of Planned Behavior to evaluate the factors influencing patient acceptance of e-health services in Saudi Arabia. Mensah (32) investigated Ghanaian citizens’ adoption of mobile health services using an extended TAM that, in addition to the core constructs of perceived usefulness and perceived ease of use, included perceived risk, mobile self-efficacy, and word-of-mouth communication. Gao et al. (1) analyzed the factors affecting the behavioral intention of enterostomy patients to use Internet diagnosis and treatment services by employing the UTAUT model alongside the Dual-factor Model. Alam et al. (33) examined the determinants of mHealth services adoption in Bangladesh using an extended UTAUT model that incorporated perceived reliability and price value factors.

In 2012, Venkatesh et al. (34) extended the UTAUT model to consumer environments by incorporating hedonic motivation, price value, and habit variables, thereby forming the Unified Theory of Acceptance and Use of Technology 2 (UTAUT2). This extension enables application of the model to a broader range of groups (e.g., users, consumers, and customers) and provides a more robust explanation of behavioral intention (BI) (35–37). Previous studies (38–43) have demonstrated that performance expectancy, effort expectancy, social influence, and price value exert significant positive effects on users' behavioral intention to adopt Internet healthcare services. Moreover, empirical investigations suggest that incorporating new and contextually relevant variables can further enhance the model's explanatory power for specific technological applications.

**Figure 1:**
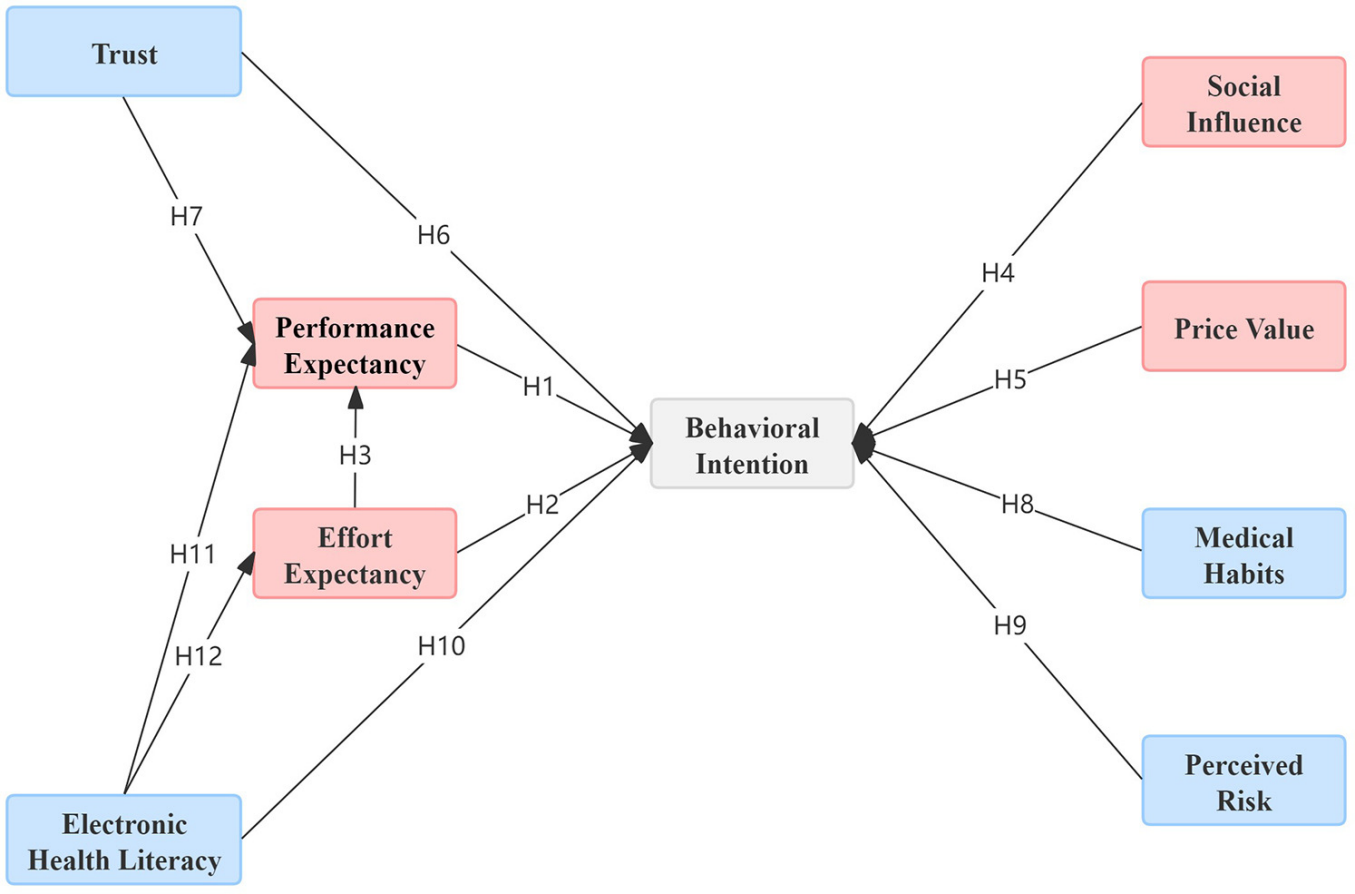
Research model. The red elements signify the fundamental variables within the theoretical framework of the UTAUT2 model, whereas the blue elements denote the supplementary variables.

To accurately and comprehensively examine the factors that influence chronic disease patients' use of Internet diagnosis and treatment services, this study utilizes the Unified Theory of Acceptance and Use of Technology 2 (UTAUT2) as its theoretical foundation. The core constructs of UTAUT2—namely, performance expectancy (PE), effort expectancy (EE), social influence (SI), and price value (PV)—are incorporated into the model. In addition, external variables, including trust (TR), medical habits (MH), perceived risk (PR), and electronic health literacy (eHL), have been introduced to extend the model. A conceptual research model has been developed (see Figure 1), which defines the relevant variables and proposes corresponding research hypotheses.

### 2.1 Performance expectancy

Performance expectancy (PE) refers to the extent to which chronic disease patients believe that using Internet diagnosis and treatment services will be beneficial (i.e., perceived usefulness). Kwateng et al. (44) reported that PE positively influences the behavioral intention of healthcare professionals to adopt and use telemedicine systems. Similarly, Rój (45) found that PE exerts the greatest impact on the acceptance and use of eHealth services among older adults in Poland, a conclusion further corroborated by Deng et al. (46). When patients perceive greater benefits in addressing their health needs through these services, their intention to use them is likely to increase. Accordingly, the following hypothesis is proposed:

H1: PE has a positive influence on the behavioral intention to use Internet diagnosis and treatment services.

### 2.2 Effort expectancy

Effort expectancy (EE) refers to the perceived ease of using Internet diagnosis and treatment services among chronic disease patients (i.e., perceived ease of use). Li et al. (47) demonstrated that EE positively influences the behavioral intention of urban older adults to use remote health management systems. Similarly, Hu et al. (48) confirmed that EE has a positive effect on the utilization of Internet healthcare services. When patients find the platform interface user-friendly and straightforward, higher EE reduces the effort required to use the service, thereby strengthening their behavioral intention. Moreover, increased EE enhances patients' perception of the usefulness of Internet diagnosis and treatment services. Accordingly, we propose the following hypotheses:

H2: EE has a positive influence on the behavioral intention to use Internet diagnosis and treatment services.

H3: EE has a positive influence on PE.

### 2.3 Social influence

Social influence (SI) refers to the extent to which chronic disease patients believe that significant others and their social environment expect them to use Internet diagnosis and treatment services. Lee et al. (49) conducted a multi-group analysis by gender and found that SI more strongly influenced female students' intention to accept telemedicine. Similarly, Nie et al. (50) demonstrated that SI positively affects older adults’ behavioral intention to use mobile healthcare services. When patients perceive that those around them and their broader social environment maintain positive and supportive attitudes toward Internet diagnosis and treatment services, their intention to adopt these services is strengthened. Accordingly, the following hypothesis is proposed:

H4: SI has a positive influence on the behavioral intention to use Internet diagnosis and treatment services.

### 2.4 Price value

Price value (PV) refers to chronic disease patients' evaluation of the service price relative to the perceived benefits of Internet diagnosis and treatment services. Wu et al. (51) confirmed that PV significantly influences the behavioral intention of older adult consumers to use smart wearable devices. Similarly, Kaur and Arora (37) found that customers' behavioral intention to use online banking services is positively affected by PV. Patients weigh the perceived benefits of diagnosis and treatment services against the actual costs incurred; when they believe that the medical assistance provided is commensurate with the costs, their intention to use the service increases. Accordingly, the following hypothesis is proposed:

H5: PV trade-offs positively influence the behavioral intention to use Internet diagnosis and treatment services.

### 2.5 Trust

Trust (TR) refers to chronic disease patients' evaluation of and attitude toward the honesty, goodwill, and competence of the services provided by Internet diagnosis and treatment platforms. This concept encompasses the professional level of healthcare providers, the quality of medical services, and internet security (52). TR is a critical factor influencing patients' use of Internet healthcare services (53). Wang et al. (54) demonstrated that higher TR fosters positive attitudes toward these platforms, thereby enhancing behavioral intention. This study posits that TR can improve patients' perception of the usefulness of Internet diagnosis and treatment services, positively influence performance expectancy (PE), and ultimately boost their behavioral intention. Similarly, Alalwan et al. (41) confirmed that TR positively influences Jordanian banking customers' PE regarding mobile banking, which in turn affects their behavioral intention. Accordingly, the following hypotheses are proposed:

H6: TR has a positive influence on the behavioral intention to use Internet diagnosis and treatment services.

H7: TR has a positive influence on PE.

### 2.6 Medical habits

Medical habits (MH) refer to the fixed behavioral patterns or preferences that individuals or groups develop when seeking medical services. These habits may be influenced by several factors, such as personal health status, economic conditions, educational background, accessibility of medical resources, and past medical experiences (55). Individuals who frequently access medical information via the Internet may develop unconscious healthcare behaviors that lead to a preference for Internet healthcare services. Chronic diseases, a category of non-communicable diseases characterized by insidious onset, prolonged course, and persistent conditions, benefit from Internet diagnosis and treatment services. These services enable patients to access medical information and professional care at any time and from any location, thus enhancing the efficiency and flexibility of healthcare delivery and fostering a tendency to use online medical resources. Consequently, this pattern of behavior promotes the adoption of Internet diagnosis and treatment services among patients with chronic diseases. Accordingly, this study proposes the following hypothesis regarding the relationships between the current variables and behavioral intention:

H8: MH has a positive influence on the behavioral intention to use Internet diagnosis and treatment services.

### 2.7 Perceived risk

Perceived risk (PR) refers to users' perception of uncertainty regarding the outcomes of using Internet diagnosis and treatment services (56). Hu et al. (57) found that when older adult users encounter issues such as personal privacy breaches or payment security risks on mobile medical platforms, they may develop resistance, thereby reducing their willingness to continue using these services. In investigating significant factors affecting patient satisfaction with Internet medical services, He et al. (58) observed that PR negatively impacts patient satisfaction. Similarly, Zhu et al. (59) demonstrated that PR adversely affects users' intention to use mobile health (mHealth) applications. Users generally tend to avoid risks; thus, when they perceive threats such as inadequate health and safety protection, privacy breaches, insecure payments, or substandard medical service quality in the context of Internet diagnosis and treatment services, their trust and reliance on these systems may decline, thereby reducing usage. Based on the foregoing theoretical analysis and existing research, the following hypothesis is proposed:

H9: PR has a negative influence on the behavioral intention to use Internet diagnosis and treatment services.

### 2.8 Electronic health literacy

Electronic health literacy (eHL) refers to an individual's ability to use electronic resources to search for, access, understand, and evaluate health information, as well as to acquire and apply health knowledge to support healthcare decisions and address health-related issues (60, 61). Wu et al. (62) indicated that eHL significantly enhances mobile health consumers' perceived ease of use and perceived usefulness, indirectly increasing their intention to use healthcare apps. Similarly, Gao et al. (1) found that patients' eHL positively affects their willingness to use Internet diagnosis and treatment services, both directly and indirectly through effort expectancy (EE). In addition, Jang (63) confirmed that eHL significantly influences the intention to use telemedicine apps by positively impacting both perceived usefulness and ease of use. Therefore, this study posits that higher eHL among patients with chronic diseases leads to greater performance expectancy (PE) and EE, which in turn enhances their behavioral intention to use Internet diagnosis and treatment services. Based on this rationale, the following hypotheses are proposed:

H10: eHL has a positive influence on the behavioral intention to use Internet diagnosis and treatment services.

H11: eHL has a positive influence on PE.

H12: eHL has a positive influence on EE.

## 3 Materials and methods

### 3.1 Questionnaire design

To ensure the validity of the research measurements, the questionnaire was developed with reference to prior studies on electronic health adoption and behavioral intention to use Internet medical services, and subsequently refined through two rounds of expert consultations. Twelve experts from multiple tertiary hospitals across Hunan, Guangdong, and Yunnan provinces were invited to participate in this study. Their expertise spans pharmaceutical information management, healthcare informatics, and medical informatics. Between March and April 2024, the questionnaire was disseminated via WeChat and email to solicit expert feedback. The relevance of each item within the various dimensions was evaluated using a four-point Likert scale. The Item-level Content Validity Index (I-CVI) was calculated as the proportion of experts rating an item's relevance as 3 or 4 out of the total number of respondents. Consultation concluded once the I-CVI for all items reached or exceeded the threshold of 0.78. Following two rounds of expert review, questionnaire items were added, deleted, or revised in accordance with experts' relevance ratings and qualitative feedback.

The revised instrument was subsequently pilot-tested, yielding 217 valid responses. Descriptive statistics indicated a predominance of male respondents (60.8%), primarily aged over 40, with most possessing undergraduate or junior college qualifications. Monthly per capita household income was concentrated between RMB 5,000 and 10,000, and the highest proportion of respondents reported hypertension. Reliability and validity analyses demonstrated excellent psychometric properties, with a Cronbach's *α* of 0.973 and a KMO measure of 0.949, thereby confirming the robustness of the scale and informing the finalization of the formal questionnaire.

The questionnaire comprised three sections: (1) General demographic information, including patients' gender, age, educational level, monthly household income, and chronic disease status; (2) Internet diagnosis and treatment service usage, including whether the service had been used, usage frequency, types of services used, and satisfaction; (3) Factors influencing the use of Internet diagnosis and treatment services, consisting of 32 items across nine dimensions corresponding to the research variables: performance expectancy (PE), effort expectancy (EE), social influence (SI), price value (PV), trust (TR), medical habits (MH), perceived risk (PR), electronic health literacy (eHL), and behavioral intention (BI). A five-point Likert scale (1 = “strongly disagree” to 5 = “strongly agree”) was used for measurement, as detailed in Table 1.

**Table 1:** Summary of construct with measurement items.

Construct	Items	Items content
Performance expectancy (PE)	PE1	I think using Internet diagnosis and treatment services can reduce the inconvenience of visiting physical hospitals and save time.
	PE2	I think using Internet diagnosis and treatment services can reduce the risk of cross-infection within hospitals.
	PE3	I think using Internet diagnosis and treatment services allows me to consult doctors more conveniently and improves the efficiency of medical consultations.
	PE4	I think using Internet diagnosis and treatment services makes it easier to obtain knowledge and information about chronic diseases.
Effort expectancy (EE)	EE1	I find the interface of Internet diagnosis and treatment services platforms user-friendly and easy to navigate.
	EE2	I find it relatively easy to perform operations related to chronic disease management on Internet diagnosis and treatment services platforms.
	EE3	I think it is very simple to learn to use Internet diagnosis and treatment services under the guidance of specialized personnel.
	EE4	I can effectively use Internet diagnosis and treatment services to meet my medical needs, such as online follow-up consultations.
Social influence (SI)	SI1	If my family, friends, or patients recommend Internet diagnosis and treatment services, I am willing to try.
	SI2	If hospitals and medical staff recommend Internet diagnosis and treatment services, I am willing to try.
	SI3	I may choose to use Internet diagnosis and treatment services under media promotion or policy guidance.
Price value (PV)	PV1	I believe the price of Internet diagnosis and treatment services is reasonable.
	PV2	I believe the cost of Internet diagnosis and treatment services is comparable to that of traditional physical hospitals.
	PV3	I believe the price of Internet diagnosis and treatment services aligns with the value they provide.
	PV4	I believe the processes and outcomes of Internet diagnosis and treatment services are the same as those of offline hospitals of the same level.
Trust (TR)	TR1	I believe that the personal information of medical staff displayed on Internet diagnosis and treatment services platforms is true and that the examination reports provided are accurate.
	TR2	I believe that the medical professionals on Internet diagnosis and treatment services platforms are qualified and capable of delivering professional healthcare services.
	TR3	I believe that the medical staff on Internet diagnosis and treatment services platforms have good attitudes and can generally meet reasonable requests and provide necessary assistance.
	TR4	I believe that the supervision of the institutions, personnel, business, quality and safety of the medical institutions carrying out Internet diagnosis and treatment activities is in line with the requirements of the regulatory rules for Internet diagnosis and treatment.
Medical habits (MH)	MH1	Before consultations, I usually use Internet diagnosis and treatment services to inquire information about doctors, diseases, and appointments.
	MH2	During consultations, I often use online consultation services to communicate with doctors about my condition and receive medication guidance.
	MH3	After consultations, I usually use Internet diagnosis and treatment services for rehabilitation guidance and health management.
Perceived risk (PR)	PR1	I am concerned about the reliability of the consultation results provided by Internet diagnosis and treatment services.
	PR2	I am concerned that using Internet diagnosis and treatment services may lead to the leakage of my personal information and medical records.
	PR3	I am concerned that the medical staff on Internet diagnosis and treatment services platforms may not be as skilled as those in physical hospitals, and misdiagnosis occurs.
Electronic health literacy (eHL)	eHL1	Obtaining health information through the Internet is of great help to me.
	eHL2	I know how to access useful health information resources on the Internet.
	eHL3	When I have health issues, I use the Internet to search for relevant information.
	eHL4	I can clearly and comprehensively provide the information required for diagnosis during online health consultations.
Behavioral intention (BI)	BI1	I am willing to learn about and use Internet diagnosis and treatment services.
	BI2	When needed in the future, I am willing to use Internet diagnosis and treatment services progressively for follow-up consultations, replacing offline hospital visits (except when physical examinations or treatments are required).
	BI3	I am willing to recommend Internet diagnosis and treatment services to others.

### 3.2 Data collection

Shenzhen is a Special Economic Zone in China with a population exceeding 10 million and is one of the most developed cities in terms of the Internet economy, boasting 27.33 million mobile Internet users (64). In 2022, cardio-cerebrovascular diseases and tumors were the first and second leading causes of death among Shenzhen residents, with 34,216 reported cases of cardiovascular diseases (an incidence rate of 194.85 per 100,000) and 42,526 reported cases of tumors (an incidence rate of 241.43 per 100,000) (65). In this megacity, the large population coupled with an unequal distribution of medical resources has become increasingly evident. Internet diagnosis and treatment services hold significant promise for addressing the needs of chronic disease management and for transforming healthcare delivery.

This study employed a cross-sectional design, targeting residents of Shenzhen, China, aged 18 years and above with a documented history of chronic diseases—including cardiovascular and cerebrovascular diseases, malignant tumors, chronic respiratory conditions, and diabetes. Data were collected between June and August 2024 through a structured questionnaire. Ethical approval was granted by the Ethics Committee of the School of Life Sciences at Central South University (Approval No. 2024-1-34). All participants provided informed consent and voluntarily engaged in the study. To ensure data integrity, preliminary screening was conducted using embedded attention-check questions. Questionnaires completed in an unrealistically short timeframe, containing missing data, or exhibiting over 80% repeated responses were subject to further exclusion. Data entry was performed independently by two researchers to guarantee accuracy. A total of 895 questionnaires were collected, of which 72 were deemed invalid, yielding 823 valid responses and an effective response rate of 91.96%.

There is no universal standard for estimating sample size across different statistical analyses; however, for structural equation modeling, a minimum sample size of 200 is generally recommended, or alternatively, a range of 5–20 times the number of items in the measurement scale is suggested (66, 67). Therefore, the number of valid questionnaires collected in this study meets the statistical criteria.

### 3.3 Statistical methods

In this study, SPSS 26.0 was used to process the survey data through descriptive statistics and to assess reliability and validity. Structural equation modeling (SEM) analysis was performed using AMOS 28.0 software for hypothesis testing and path analysis of the research model. A *p*-value of less than 0.05 was considered statistically significant.

## 4 Results

### 4.1 Descriptive analysis of demographic characteristics

Among the 823 respondents, 51.4% were male and 48.6% were female. Most participants were between 18 and 49 years old (79.7%). In terms of education, 67.8% had attained an undergraduate or junior college degree. The per capita monthly family income primarily ranged from 5,000 to 15,000 yuan (79.1%). Additionally, 46.9% of participants had one or more chronic conditions, with hypertension (50.8%), chronic bronchitis (39.6%), and diabetes (39.5%) being the most common, as detailed in Table 2.

**Table 2:** Demographic characteristics of respondents.

Item	Categories	Frequency (*N* = 823)	Percentage (%)
Gender	Male	423	51.4
	Female	400	48.6
Age	18–29	181	22.0
	30–39	294	35.7
	40–49	181	22.0
	50–59	117	14.2
	60 and above	50	6.1
Educational level	Junior high school and below	30	3.6
	High school/technical secondary school	190	23.1
	Undergraduate/Junior College	558	67.8
	Master's degree and above	45	5.5
Family monthly income	Below 5,000 RMB	60	7.3
	5,000–10,000 RMB	401	48.7
	10,001–15,000 RMB	250	30.4
	Above 15,000 RMB	112	13.6
Chronic disease status	Hypertension	418	50.8
	Diabetes	325	39.5
	Coronary heart disease	150	18.2
	Chronic bronchitis	326	39.6
	Chronic obstructive pulmonary disease	99	12.0
	Other chronic diseases	14	1.7

### 4.2 Use of internet diagnosis and treatment services

Of the 823 respondents, 785 (95.4%) reported having used Internet diagnosis and treatment services. The most common usage frequency was once per month (35.7%), followed by once every 2 weeks (27.0%). Usage rates for services such as appointment booking, online consultations, and health consultations all exceeded 70%. Additionally, 58.0% of respondents expressed high satisfaction with these services, while 39.4% were relatively satisfied. Among the 38 participants who had never used these services, the primary reasons were lack of trust (65.8%), uncertainty about how to use them (50.0%), and concerns regarding security and privacy (42.1%), as detailed in Table 3.

**Table 3:** Use of Internet diagnosis and treatment services of respondents.

Item	Categories	Frequency (*N* = 823)	Percentage (%)
Use experience	Yes	785	95.4
	No	38	4.6
Usage frequency	Weekly	113	14.4
	Half a month	212	27.0
	One month	280	35.7
	Three months	120	15.3
	Half a year	50	6.4
	Rarely used	10	1.3
Used service types	Appointment booking	594	75.7
	Health consultation	569	72.5
	Online consultation	570	72.6
	Issuance of electronic prescriptions	251	32.0
	Prescription renewal for chronic diseases	178	22.7
	Other services	0	0
Satisfaction	Very satisfied	455	58.0
	Quite satisfied	309	39.4
	Neutral	17	2.2
	Quite dissatisfied	4	0.5
	Very dissatisfied	0	0
Primary reasons for not using	Lack of knowledge about how to use Internet diagnosis and treatment services	19	50.0
	Preference for face-to-face consultations and lack of trust in Internet diagnosis and treatment services	25	65.8
	Concerns about the security and privacy protection of Internet diagnosis and treatment services	16	42.1
	Concerns that the medical quality of Internet diagnosis and treatment services is not as good as traditional services	10	26.3
	Concerns that Internet diagnosis and treatment services cannot meet personal health needs	7	18.4
	Other reasons	0	0

### 4.3 Reliability and validity analysis

Cronbach's alpha was employed to assess scale reliability, with coefficients above 0.7 indicating acceptable reliability. Convergent validity was evaluated using standardized factor loadings, average variance extracted (AVE), and composite reliability (CR), whereby factor loadings above 0.6, an AVE exceeding 0.5, and a CR greater than 0.7 indicate good convergent validity (68). As shown in Table 4, the Cronbach's alpha coefficients for the latent variables ranged from 0.851 to 0.865, the standardized factor loadings from 0.745 to 0.865, the AVE from 0.590 to 0.683, and the CR from 0.851 to 0.866. These results confirm that the questionnaire met internal consistency reliability requirements and demonstrated good convergent validity.

**Table 4:** Results of reliability and validity.

Variables	Items	Loading	Cronbach's alpha	AVE	CR
Performance expectancy (PE)	PE1	0.812	0.857	0.603	0.858
	PE2	0.774			
	PE3	0.745			
	PE4	0.773			
Effort expectancy (EE)	EE1	0.782	0.852	0.591	0.852
	EE2	0.767			
	EE3	0.766			
	EE4	0.759			
Social influence (SI)	SI1	0.812	0.851	0.656	0.851
	SI2	0.794			
	SI3	0.824			
Price value (PV)	PV1	0.809	0.855	0.596	0.855
	PV2	0.757			
	PV3	0.759			
	PV4	0.762			
Trust (TR)	TR1	0.767	0.861	0.607	0.861
	TR2	0.801			
	TR3	0.765			
	TR4	0.784			
Medical habits (MH)	MH1	0.847	0.856	0.666	0.857
	MH2	0.803			
	MH3	0.797			
Perceived risk (PR)	PR1	0.822	0.865	0.683	0.866
	PR2	0.817			
	PR3	0.840			
Electronic health literacy (eHL)	eHL1	0.808	0.852	0.590	0.852
	eHL2	0.751			
	eHL3	0.757			
	eHL4	0.756			
Behavioral intention (BI)	BI1	0.814	0.856	0.668	0.858
	BI2	0.770			
	BI3	0.865			

Discriminant validity for each latent variable was evaluated by comparing the square root of the average variance extracted (AVE) with the correlation coefficients among variables. Specifically, if the square root of a variable's AVE exceeds its correlations with other variables, it indicates adequate discriminant validity (68). The results in Table 5 confirm that the latent variables in the measurement model exhibit robust discriminant validity.

**Table 5:** Matrix of correlation constructs and discriminant validity.

Variable	PE	EE	SI	PV	TR	MH	PR	eHL	BI
PE	**0** **.** **777**								
EE	0.614	**0** **.** **769**							
SI	0.423	0.375	**0** **.** **810**						
PV	0.478	0.474	0.450	**0** **.** **772**					
TR	0.402	0.355	0.405	0.501	**0** **.** **779**				
MH	0.418	0.440	0.322	0.330	0.295	**0** **.** **816**			
PR	−0.294	−0.253	−0.272	−0.273	−0.275	−0.166	**0** **.** **826**		
eHL	0.461	0.372	0.484	0.402	0.338	0.406	−0.284	**0** **.** **768**	
BI	0.518	0.464	0.524	0.501	0.452	0.331	−0.337	0.505	**0** **.** **817**

The bold values on the diagonal represent the square roots of the AVE for the respective constructs, while the off-diagonal values indicate the correlation coefficients between the latent variables.

### 4.4 Evaluation of structural equation models

Structural equation modeling was conducted using AMOS software to analyze the collected questionnaire data and evaluate the model's goodness-of-fit. Detailed results of the fit indices are presented in Table 6. The comprehensive fit statistics fall within acceptable thresholds, indicating a satisfactory fit of the structural equation model.

**Table 6:** Model Fit indices for the structural equation model.

Fit indices	*χ*^2^/df	RMSEA	GFI	AGFI	NFI	IFI	CFI
Ideal criteria	<3	<0.08	>0.9	>0.9	>0.9	>0.9	>0.9
Model results	1.702	0.029	0.947	0.936	0.947	0.977	0.977

**Figure 2:**
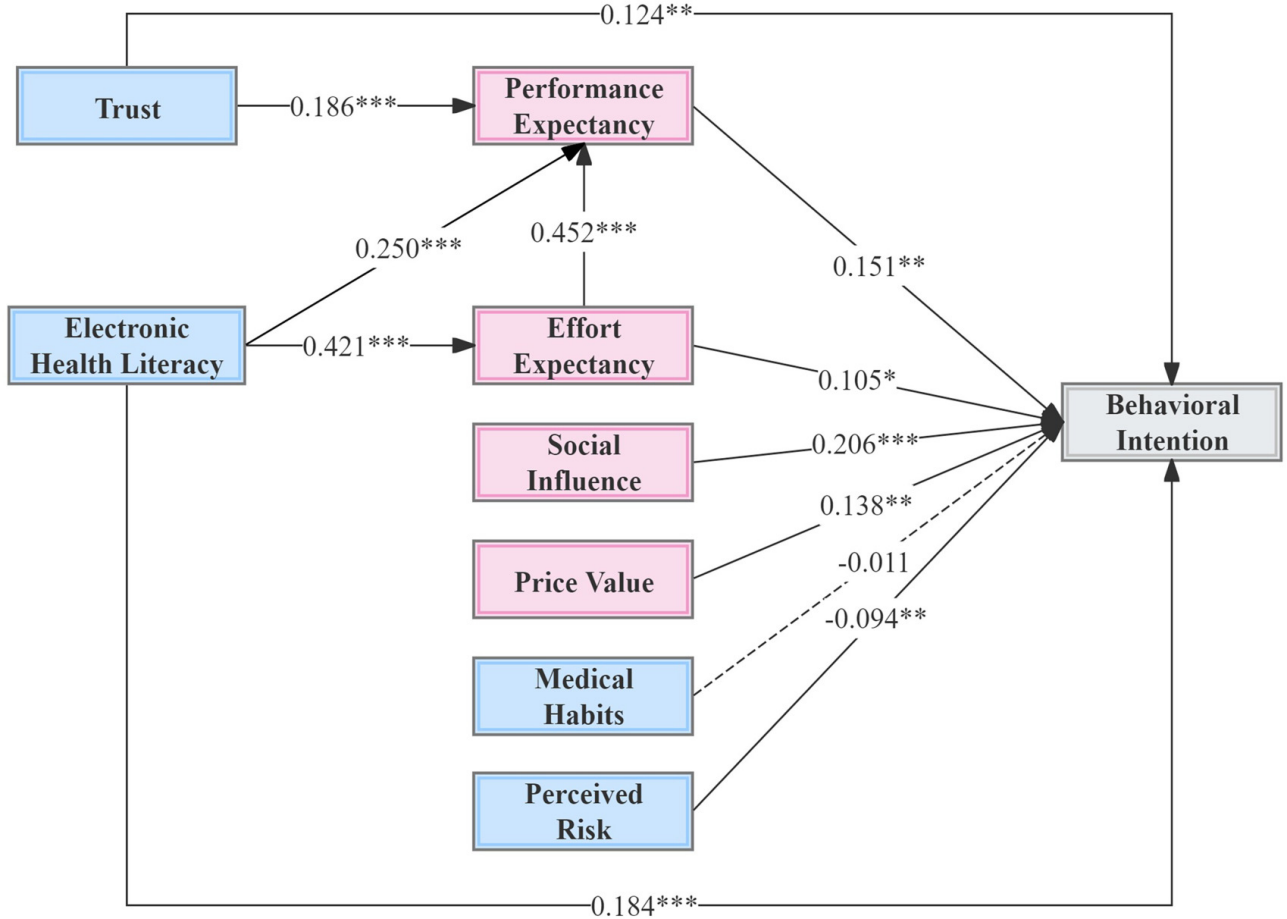
Structural model results. *p < 0.05, **p < 0.01, ***p < 0.001; and the dotted line represents insignificant path.

The structural model was developed to examine the relationships among the hypothesized constructs. Hypothesis testing for the theoretical model was conducted using standardized coefficients (*β*) and *t*-statistics for the paths in the structural equation model. The standardized path coefficients and their significance levels for each hypothesized path are presented in Figure 2 and Table 7. The results indicate that performance expectancy (PE; *t* = 3.123, *β* = 0.151, *p* < 0.01), effort expectancy (EE; *t* = 2.282, *β* = 0.105, *p* < 0.05), social influence (SI; *t* = 4.774, *β* = 0.206, *p* < 0.001), price value (PV; *t* = 3.133, *β* = 0.138, *p* < 0.01), trust (TR; *t* = 2.965, *β* = 0.124, *p* < 0.01), and electronic health literacy (eHL; *t* = 3.991, *β* = 0.184, *p* < 0.001) all exerted significant positive influences on behavioral intention. Conversely, perceived risk (PR; *t* = −2.650, *β* = −0.094, *p* < 0.01) had a significant negative impact, while the effect of medical habits (MH) was not statistically significant. Consequently, hypotheses H1, H2, H3, H4, H5, H6, H7, H9, H10, H11, and H12 were supported, whereas H8 was not supported in this study.

**Table 7:** Hypothesis testing results of the research model.

Hypothesis	Path	*β*	S.E.	*t*-statistics	*p*-value	Results
H1	PE → BI	0.151	0.055	3.123	0.002	Accepted
H2	EE → BI	0.105	0.047	2.282	0.022	Accepted
H3	EE → PE	0.452	0.038	10.758	***	Accepted
H4	SI → BI	0.206	0.044	4.774	***	Accepted
H5	PV → BI	0.138	0.047	3.133	0.002	Accepted
H6	TR → BI	0.124	0.045	2.965	0.003	Accepted
H7	TR → PE	0.186	0.035	4.997	***	Accepted
H8	MH → BI	−0.011	0.037	−0.281	0.779	Rejected
H9	PR → BI	−0.094	0.029	−2.650	0.008	Accepted
H10	eHL → BI	0.184	0.052	3.991	***	Accepted
H11	eHL → PE	0.250	0.040	6.214	***	Accepted
H12	eHL → EE	0.421	0.046	10.109	***	Accepted

*** *p* < 0.001.

The Bootstrap method was employed to examine the mediating effects in the model. A total of five mediating effects were hypothesized in this study, as follows: int1: EE → PE → BI; int2: TR → PE → BI; int3: eHL → PE → BI; int4: eHL → EE → PE → BI; int5: eHL → EE → BI. Through hypothesis testing and path analysis, five mediating effects in the model exist.

## 5 Discussion

This study applied the UTAUT2 model to examine chronic disease patients' behavioral intentions to adopt Internet diagnosis and treatment services. The findings provide empirical support for the hypotheses concerning PE, EE, SI, and BI within the acceptance and usage framework of these services, thereby extending the application of UTAUT2 in this domain. The results indicate that PE, EE, SI, PV, TR, PR, and eHL are the primary factors influencing chronic disease patients’ behavioral intentions to use these services, whereas MH was not significant. A discussion of these influencing factors follows.

PE, EE, SI, and PV significantly and positively influenced chronic disease patients' behavioral intentions to use Internet diagnosis and treatment services, a finding that aligns with previous research both domestically and internationally. Nisha et al. (69) identified EE, PE, and SI as significant factors affecting Bangladeshi consumers' intentions to adopt mobile health services. Similarly, Macedo (70) demonstrated in an empirical study on the acceptance of information and communication technology (ICT) among Portuguese older adults that PE, EE, and SI positively impact behavioral intentions to use ICT. In addition, a study examining the continuance intentions of elders with chronic diseases regarding mobile health services (71) confirmed the significant positive effects of these variables.

PE positively influences behavioral intentions, suggesting that chronic disease patients are more willing to use Internet diagnosis and treatment services when they perceive these services as beneficial for addressing shortcomings in traditional healthcare. Therefore, Internet diagnosis and treatment platforms can optimize diagnostic and treatment processes by offering services such as time-slot–based appointment scheduling and intelligent triage for medical guidance. Moreover, strengthening the integration between online and offline healthcare services can further enhance the effectiveness of Internet hospitals and increase patients' performance expectancy.

EE not only exerts a direct positive influence on behavioral intentions but also indirectly enhances these intentions through the mediating effect of PE. This mediating effect is consistent with findings from a study on mobile healthcare services for the older adult in Shanghai (72). EE can be improved by enhancing the ease of learning, simplicity, and operability of the service. For instance, streamlining processes for online registration, medical consultations, medication purchases, and payment transactions—as well as designing clear, intuitive user interfaces that allow patients to quickly locate desired functions—can enhance EE. Moreover, addressing the specific needs of older adult patients by optimizing platforms for age-friendliness is crucial. Such optimizations may include introducing a “care mode” featuring adjustable font sizes, highlighted keyword buttons, simplified operational processes, and additional functions such as voice input and touch-to-read options.

SI positively influences behavioral intentions, indicating that external perceptions—such as the attitudes and opinions of family members, friends, healthcare professionals, and policy guidance—affect chronic disease patients' willingness to use Internet diagnosis and treatment services. This finding is consistent with the conclusions of Wang et al. (4) and Barua et al. (73). Therefore, in addition to using both online and offline media for promotion, strategies should also focus on disseminating positive information through social networks to enhance patient engagement and improve their awareness and acceptance of Internet diagnosis and treatment services.

PV trade-offs positively influence behavioral intentions, which aligns with the findings of Ravangard et al. (74). In a study examining the primary factors affecting patients’ willingness to use “Internet Plus” Traditional Chinese Medicine services, Tian (75) identified PV as the most significant factor. Patients consider various factors—including treatment costs, price comparisons between online and offline services, and health insurance reimbursement—when utilizing Internet diagnosis and treatment services. Therefore, these platforms should enhance collaboration with government agencies and medical insurance departments to secure policy support, facilitate reimbursement, and establish detailed payment standards for services covered by medical insurance.

TR positively influences PE and behavioral intentions, which is consistent with previous research findings. Specifically, when chronic disease patients have higher levels of trust in Internet diagnosis and treatment services, they are more likely to perceive these services as beneficial, thereby enhancing their behavioral intentions. Jiang et al. (76) found that users' trust in mobile e-Health information services positively impacted their willingness to use these services. Similarly, Zhu et al. (59) reported that perceived trust positively affects users’ intentions to use mobile health applications, and Ekaimi et al. (53) demonstrated that TR positively impacts patients' behavioral intentions to use telemedicine services. A study on the continued use of mobile healthcare services among the older adult (57) corroborated these findings. Furthermore, our questionnaire survey revealed that a lack of trust is one of the primary reasons patients do not use Internet diagnosis and treatment services. To address this issue, platforms should establish a strong brand image, strictly regulate resident doctors, and clearly display doctors' qualification certificates, professional backgrounds, and treatment experience. In addition, exaggerated advertisements should be minimized to enhance patients' trust and acceptance of Internet diagnosis and treatment services.

PR negatively influences behavioral intentions. Chronic disease patients' perceived risks—such as network security issues, information leakage, and uncertainty about service quality—diminish their willingness to use Internet diagnosis and treatment services. This finding is consistent with the conclusions of Chen et al. (77) and Deng et al. (78). Nie et al. (5) surveyed patients with hypertension and diabetes and found that higher levels of perceived risk in mobile healthcare services are associated with lower willingness to adopt them. Similarly, Zhang et al. (79) confirmed that perceived privacy risks negatively impact patients' intentions to use diabetes management apps. Consequently, it is imperative to address and mitigate these risks by continuously optimizing the technical architecture and security of Internet diagnosis and treatment platforms. Robust data encryption should be implemented to ensure that patient data are adequately protected during transmission and storage, thereby preventing data leakage or unauthorized access. Furthermore, regular security audits and vulnerability scans should be conducted to strengthen system defenses.

eHL positively influences behavioral intentions, consistent with findings from numerous studies (80–82) both domestically and internationally. Wang et al. (83) demonstrated that older adult individuals with smartphones and proficient operational skills better adapt to the Internet healthcare model. A survey of older adult patients with chronic diseases revealed that those with higher eHL are more likely to use Internet diagnosis and treatment services for health consultation and management (84). Similarly, Yadana et al. (85) found that higher eHL is associated with better self-perceived health and more frequent use of digital technologies for accessing health and care services. Moreover, eHL exerts both direct and indirect positive effects on behavioral intentions through EE and PE. Patients with higher eHL can quickly learn and master the operational processes of Internet diagnosis and treatment services, thereby reducing usage difficulty and cost while enhancing perceived usefulness. This increased ease of use and adaptability further strengthens their willingness to adopt these services.

This study found that the path coefficient for the influence of MH (Medical Habits) on the willingness to use Internet diagnosis and treatment services among chronic disease patients did not reach a statistically significant level. Consequently, the theoretical hypothesis H8 was not supported, indicating that habitual reliance on online medical resources does not significantly promote patients' adoption of Internet diagnosis and treatment services. This finding aligns with the results of several scholars' studies. For instance, Liu's research (86) revealed that the negative impact of patients' offline healthcare habits on their willingness to use Internet diagnosis and treatment services was not significant, a finding that is consistent with those of Ravangard et al. (74) and Alam et al. (87), who likewise reported an insignificant relationship between habits and willingness to use Internet healthcare services. This may be attributable to the fact that healthcare services are directly linked to patients’ health and well-being. When making related decisions, patients typically exhibit higher levels of caution. The Health Belief Model (88) underscores the pivotal role of perception in decision-making, positing that a constellation of factors shapes these perceptions, which in turn influence the likelihood of adopting health-promoting behaviors. Chronic disease management often requires the coordination of both online and offline services, prompting patients to consider a broader range of factors such as disease type, severity, and treatment complexity when selecting healthcare services. These mediating factors may indirectly attenuate the relative influence of habitual behavior. Consequently, the direct influence of patients' established medical habits on their behavioral intentions to adopt Internet diagnosis and treatment services appears to be relatively minimal.

## 6 Limitations and future research directions

Grounded in the UTAUT2 theoretical framework, this study incorporated external variables—namely, Trust, Medical Habits, Perceived Risk, and Electronic Health Literacy—that may influence technology acceptance, as identified through a literature review. Although this approach provides a more comprehensive perspective, it has certain limitations, including constraints inherent to the theoretical framework, methodological restrictions, and sample representativeness. Constrained by temporal and human resource limitations, this study exclusively conducted a cross-sectional investigation of the core variables, omitting the examination of moderating factors and thereby failing to elucidate how the relationships among key constructs may vary across different populations or contextual conditions. Future research should incorporate potential moderators, such as age, gender, education level, and family income, within a longitudinal assessment to better elucidate the mechanisms underlying chronic disease patients' behavioral intentions to use Internet diagnosis and treatment services. Such an approach would enable a dynamic understanding of behavioral change over time and illuminate the evolving complexity of intervariable relationships.

Moreover, the sample was drawn exclusively from Shenzhen, a special economic zone in China, which may limit the generalizability and applicability of the findings. Given the region's highly developed digital economy, residents may exhibit greater accessibility to and familiarity with Internet diagnosis and treatment services compared to populations in less economically developed areas, potentially introducing contextual bias. Future studies should expand the sample scope to enhance the universality of the results. In addition, more in-depth analyses targeting subpopulations defined by age, educational attainment, digital literacy levels, and health-related needs may further elucidate both the heterogeneity and commonalities in behavioral intention to use Internet diagnosis and treatment services across diverse demographic groups.

Finally, this study examined behavioral intentions solely from the perspective of chronic disease patients without investigating actual usage behavior. Future research could integrate multiple perspectives—including those of healthcare professionals and family members—and employ diverse data collection methodologies. For instance, in-depth interviews may uncover stakeholders' subjective motivations and attitudes, such as the psychological determinants underpinning patient adherence, while ethnographic observation could identify objective barriers encountered in real-world usage contexts, such as technical challenges within operational workflows, and conduct both observational and statistical analyses of patients' actual usage behaviors. Drawing upon the intention-behavior gap theoretical framework, this study further investigates the mechanisms through which behavioral intention translates into actual usage behavior.

## 7 Conclusion

Although Internet diagnosis and treatment services hold significant promise in China, harnessing their full potential requires a deep understanding of the factors driving user demand and engagement. In a novel approach, this study explores the attitudinal determinants influencing chronic disease patients' acceptance and use of these services—an area that remains underexamined in current research. By employing an innovative framework that integrates key constructs—PE, EE, SI, PV, TR, and eHL—with a focus on reducing PR, our empirical findings reveal that enhancing these factors can substantially boost patients' behavioral intentions. This work not only provides practical guidelines for implementing Internet healthcare services but also offers valuable insights for the design and development of Internet diagnosis and treatment platforms in developing countries, thereby making a significant and innovative contribution to the field.

Existing laws, regulations, and policy documents are inadequate to comprehensively and effectively guide and regulate the current development of Internet healthcare services. As a result, the urgent need to improve the related supportive policies and systems must be addressed. On one hand, clear and explicit policy support could be introduced to expand the social impact from the government level, integrating resources from sectors such as public health, medical insurance, and market regulation. This would promote the standardization and widespread adoption of Internet diagnosis and treatment services, providing legal protections for both healthcare providers and patients. On the other hand, a long-term, dynamic regulatory mechanism should be established to comprehensively supervise all aspects of Internet diagnosis and treatment service platforms, including service quality, data security, and privacy protection. Furthermore, a well-defined certification system for healthcare providers must be implemented, ensuring that the fees for diagnosis and treatment are commensurate with the services provided, with transparent billing standards, all while safeguarding patient rights.

Internet diagnosis and treatment service platforms should meaningfully consider the actual needs of patients, enrich online diagnostic resources, and enhance the quality and functional utility of the services provided. At the same time, it is critical to ensure that healthcare professionals offering Internet diagnosis and treatment services possess valid professional qualifications and extensive clinical experience. Special attention should be paid to the professional skills and service attitudes of these personnel, and regular professional training and performance evaluations should be organized to ensure they remain up-to-date with the latest medical advancements, thereby improving their professional competence. Additionally, stronger integration with offline medical institutions is essential to facilitate data sharing and integration across healthcare providers, thereby establishing an integrated online-offline healthcare service system that encompasses the full healthcare lifecycle—pre-diagnosis, diagnosis, and post-diagnosis care.

## Data Availability

The raw data supporting the conclusions of this article will be made available by the authors, without undue reservation.
